# Correction: A digital microscope for the diagnosis of *Plasmodium falciparum* and *Plasmodium vivax*, including *P. falciparum* with *hrp2/hrp3* deletion

**DOI:** 10.1371/journal.pgph.0007614

**Published:** 2026-11-10

**Authors:** Yalemwork Ewnetu, Kingsley Badu, Lise Carlier, Claudia A. Vera-Arias, Emma V. Troth, Abdul-Hakim Mutala, Stephen Opoku Afriyie, Thomas Kwame Addison, Nega Berhane, Wossenseged Lemma, Cristian Koepfli

After publication of this article [1], the following errors were identified in Table 1 and the article text:

- In Table 1A, the Ghana RDT (HRP2/LDH) Sensitivity at 200 parasites/µL contains a typographical error. The correct value is 94.6% (175/185).
- In Table 1A, the Combined Microscopy values contain errors in the recorded sensitivity percentages and are erroneously included in the published table. The authors note that as the protocol to obtain these data differed between the two sites, they should not have been combined and they have therefore been removed from the revised Table 1.
- In the Sensitivity and specificity of *P*. *falciparum* and *P*. *vivax* diagnosis subsection of the Results section, there is an error in the specificity of miLab in the penultimate sentence of the third paragraph. The correct sentence is: Only four samples were determined positive for *P. vivax* by miLab but negative for either species by qPCR, resulting in a specificity of 97.6%.

With this Correction, the authors also provide additional and updated methodological information on the sensitivity and specificity calculations outlined below, in relation to each diagnostic test and presence of single or multiple malaria species.

For data from Ethiopia, where *P. falciparum* and *P. vivax* are co-endemic, sensitivity for *P. falciparum* for the miLab automated microscope was calculated as the number of samples positive by miLab for either species (i.e., *P. falciparum* or *P. vivax*) divided by number positive for *P. falciparum* by qPCR. Thus, true positives are defined as samples positive by miLab for *P. falciparum* or *P. vivax*, and false negatives are defined as samples negative by miLab but positive for *P. falciparum* by qPCR. Sensitivity for *P. vivax* for the miLab automated microscope was calculated as the number of samples positive by miLab for either species (i.e., *P. falciparum* or *P. vivax*) divided by number positive for *P. vivax* by qPCR. Thus, true positives are defined as samples positive by miLab for *P. falciparum* or *P. vivax*, and false negatives are defined as samples negative by miLab but positive for *P. vivax* by qPCR. For the current study, infections at densities <20 parasites/µL were excluded from sensitivity calculations to aid comparison with other studies.
Specificity for either species for a given diagnostic test was calculated as true negatives/ (true negatives + false positives). For *P. falciparum* diagnosis, true negatives are defined as samples negative by a diagnostic test for *P. falciparum* and negative by qPCR for both species (i.e., negative for *P. falciparum* and negative for *P. vivax*), and false positives are defined as positive for *P. falciparum* by a diagnostic test but negative by qPCR for both species (i.e., negative for *P. falciparum* and negative for *P. vivax*). For *P. vivax* diagnosis, true negatives are defined as samples negative by a diagnostic test for *P. vivax* and negative by qPCR for both species (i.e., negative for *P. falciparum* and negative for *P. vivax*), and false positives are defined as positive for *P. vivax* by a diagnostic test but negative by qPCR for both species (i.e., negative for *P. falciparum* and negative for *P. vivax*).

Following this definition, the authors updated the specificity calculation for the rapid diagnostic test (RDT) to be restricted to samples negative by qPCR for both species to be consistent with the specificity calculations for detection by microscopy and miLab.

With the updated methodological information outlined above, the following updates are made to the Study population and methods section and Table 1:

- In the Data analysis subsection of the Study population and methods section, the diagnostic methods listed in the sixth sentence of the third paragraph are updated to: In line with this, calculations of specificity for all diagnostic methods were restricted to samples negative by qPCR for either species.
- In Table 1A, the Ethiopia RDT (HRP2) Specificity value in brackets is now calculated with only samples negative by qPCR for either species. The updated value is 100% (164/164).
- In Table 1, the second sentence in the text given underneath the table does not specify the type of positive or negative samples in the brackets. The updated sentence is: For sensitivity results, numbers in brackets show the number of true positives over the total number of positive samples exceeding a given parasite density (by qPCR). For specificity results, the numbers in brackets show the number of true negatives over the total number of samples negative (by qPCR) for both species (i.e., negative for *P. falciparum* and negative for *P. vivax*).

Please see the complete and correct Table 1 provided with this Correction. A member of the *PLOS Global Public Health* Editorial Board confirmed that the updates to Table 1 do not affect the conclusions of [1].

**Table 1:** Sensitivity and specificity for the diagnosis of *P. falciparum* and P*. vivax* compared to qPCR as gold standard.

A) *P. falciparum*				
	Sensitivity at 200 parasites/µL	Sensitivity at 20 parasites/µL	Specificity	AUC > 20 parasites/µL
	**Ethiopia**			
miLab	98.0% (145/148)	96.3% (154/160)	86.0% (141/164)	86.30%
Microscopy^1^	83.1% (123/148)	78.1% (125/160)	99.4% (163/164)	87.00%
RDT (HRP2)^2^	89.5% (85/95)	86.7% (85/98)	100% (164/164)	78.40%
				
	**Ghana**			
miLab	91.4% (169/185)	74.6% (188/252)	96.3% (567/589)	85.40%
Microscopy^1^	83.8% (155/185)	64.7% (163/252)	98.5% (580/589)	81.60%
RDT (HRP2/LDH)^3^	94.6% (175/185)	84.1% (212/252)	98.0% (577/589)	91.00%
				
	**Combined**			
miLab	94.3% (314/333)	83.0% (342/412)	94.0% (708/753)	86.30%
				
**B) *P. vivax***				
	Sensitivity at 200 parasites/µL	Sensitivity at 20 parasites/µL	Specificity	AUC > 20 parasites/µL
miLab	97.0% (65/67)	93.9% (77/82)	97.6% (160/164)	87.10%
Field microscopy	80.1% (54/67)	67.0% (55/82)	97.0% (159/164)	81.30%
RDT	91.0% (61/67)	76.8% (63/82)	97.6% (160/164)	87.30%

All density thresholds are based on qPCR data. For sensitivity results, numbers in brackets show the number of true positives over the total number of positive samples exceeding a given parasite density (by qPCR). For specificity results, the numbers in brackets show the number of true negatives over the total number of samples negative (by qPCR) for both species (i.e., negative for *P. falciparum* and negative for *P. vivax*).

^1^ In Ethiopia, microscopy data from the health center was recorded (i.e., no expert microscopy was conducted). In Ghana, expert microscopy data was recorded.

^2^ Samples with *hrp2* deletion were excluded from calculations.

^3^ Any RDT positive either for LDH or HRP2 was considered positive.
