# Supplementary figures and images for: A digital microscope for the diagnosis of Plasmodium falciparum and Plasmodium vivax, including P. falciparum with hrp2/hrp3 deletion
Source: PLOS Glob Public Health. 2024 May 20;4(5):e0003091. doi: 10.1371/journal.pgph.0003091 (PMC11104649; doi:10.1371/journal.pgph.0003091)

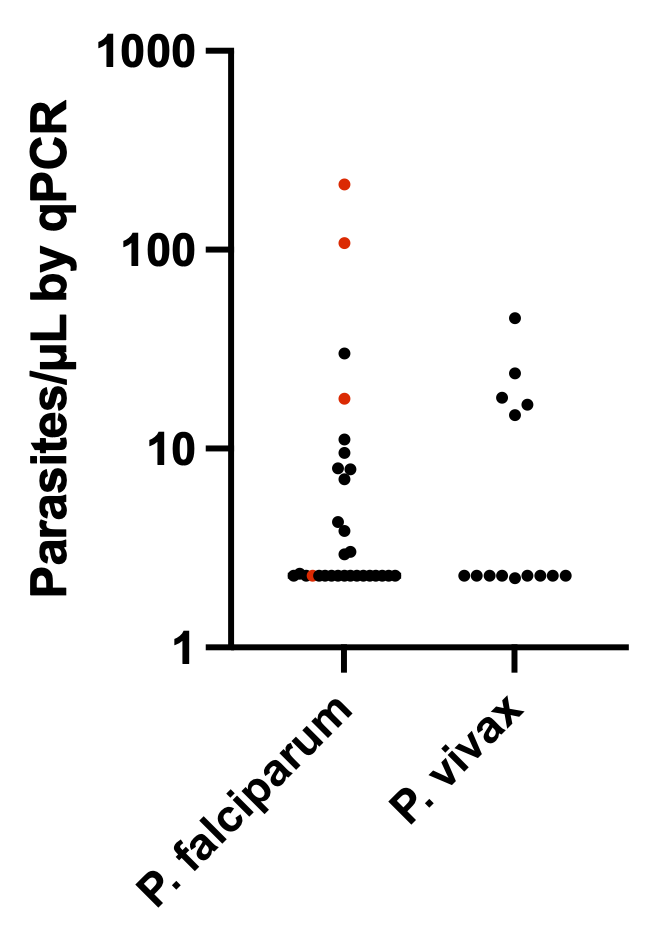

Supplement: S1 Fig — Samples found positive by miLab are indicated in red. (TIFF) [file pgph.0003091.s003.tiff]
