# Supplementary material for: A digital microscope for the diagnosis of Plasmodium falciparum and Plasmodium vivax, including P. falciparum with hrp2/hrp3 deletion
Source: PLOS Glob Public Health. 2024 May 20;4(5):e0003091. doi: 10.1371/journal.pgph.0003091 (PMC11104649; doi:10.1371/journal.pgph.0003091)
Supplement: Attachment — Submitted filename: Koepfli et al reports.doc [file pgph.0003091.s005.doc]

Koepfli et al reports ‘A digital microscope for the diagnosis of Plasmodium falciparum and Plasmodium vivax, including P. falciparum with hrp2/hrp3 deletion’. Authors report malaria diagnostic challenges especially where multiple Plasmodium species are endemic. To address such challenges, the performances of a digital automated portable digital microscope from Noul MiLab were evaluated among 659 suspected malaria patients in Gondar, Ethiopia and 991 patients in Ghana. According to authors report miLab shows superior performance as compared to microscopy and comparable performance with RDTs. Authors also concluded that miLab was more sensitive than microscopy and thus is a valuable addition to the toolkit for malaria diagnosis, particularly for areas with high frequencies of hrp2/3 deletions.

**Comments**

1. The details of the performance of digital microscopy for the diagnosis of falciparum malaria in Ethiopia and Ghana were not well presented.
2. Authors didn’t indicate which specific regions of the hrp2/3 loci (exon 2, exon 1-2, upstream or downstream flanking regions) were genotyped to determine hrp2/3 gene deletion.
3. It’s unclear whether Noul MiLab, MiLab and digital microscope are interchangeably used as each appears at various places.
4. The specificity of the miLab was 94.0%, which indicates the presence of ‘false positive’ results.
5. The same RDTs were not used in Ethiopia and Ghana, hence, comparison of the RDT sensitivity seems challenging between the 2 countries.
6. Under MM section (line 116-117), it reads that ‘*All samples were tested on site by miLab, and RDT*’. I’m wondering why microscope wasn’t used onsite for malaria diagnosis given the study site is only 2,100meters away from Gondar town of Ethiopia?. Is this for Ghana or Ethiopia? Does the national policy of Ethiopia support this?
7. In line 180-181, it reads that ‘If a sample was positive by qPCR for P. falciparum but the miLab diagnosed possible P. vivax (or vice versa), it was counted as positive’. In country like Ethiopia where Pf and Pv are the two plasmodium species that co-exist, species identification is critical as the country has species-specific treatment policy. Does this give sense as far as miLab-based malaria diagnosis is concerned in Ethiopia?
8. Under data analysis (line 182-183), it reads that ‘the number of infections where the incorrect species was diagnosed, or mixed-species infections with only one species diagnosed, are presented separately’. It’s unclear why such data analysis is considered and authors shall provide details. The data analysis has to be made for all the infections regardless of whether they are correctly diagnosed or not.
9. How authors did select the study sites in Ethiopia and Ghana?
10. Did authors enroll every febrile patient who sought malaria diagnosis at both health centers of Ethiopia and Ghana? How authors account for the variation in sensitivity of DBS-based qPCR (in Ethiopia) vs whole-blood based PCR (Ghana) for malaria diagnosis?
11. It is known that malaria is more endemic to Ghana than Ethiopia; however, it appears that the prevalence of malaria looks higher in Ethiopia (75.1% (495/659) individuals tested positive) than in Ghana (40.6% 402/991) tested positive) for P. falciparum. How authors justify this?
12. Line 209-212, under ‘Sensitivity and specificity of P. falciparum and P. vivax diagnosis’. Is it possible for the authors to calculate the combined sensitivity of miLab (94.3% at densities >200 parasites/µL) for both Ethiopia and Ghana given Pv is uncommon in the latter? Or is the sensitivity of miLab for Pf only?
13. Although the sensitivity and specificity of miLab were calculated as shown in table 1; it’s unclear which tool(s) (qPCR or microscopy) was taken as a gold standard. Or do authors think/believe that miLab is superior to qPCR?
14. It would be important to describe the total number of Pf and Pv samples subjected to calculate miLab sensitivity/specificity in table 1 so that readers can easily appreciate its diagnostic performances.
15. The specificity of microscope and RDT is superior to that of miLab (table 1) in Ethiopia, how authors justify that miLab could lead to the treatment of uninfected individuals with antimalarial drugs? i.e from drug pressure, or drug resistance emergence/development perspectives?
16. Line 241-245, it’s indicated that ‘the miLab successfully diagnosed 51/52 (98.1%) infections with *hrp2* deletion while field microscopy diagnosed 37/52 (71.2%) of *hrp2*-negative infections; what justification could authors provide for the inability of the microscope to detect of hrp2-deleted parasites while miLab did? Authors shouldn’t underestimate the issue of Pv misdiagnosis (5 infections) in Ethiopia.
17. Line 238, when authors present ‘Diagnosis of hrp2/3 negative P. falciparum infections’, it would be imperative if they also provide ‘Diagnosis of hrp2/3 positive P. falciparum infections’ for readers to appreciate it.
18. Line 249-253, authors report that miLab has severe shortcomings in accurately diagnosing mixed infections (either the wrong species was assigned, or only one species was diagnosed in mixed species infections). Thus, in setting like Ethiopia where Pf and Pv are common and species-specific treatment policy exists, miLab diagnosis would lead to wrong drug regimens. Nevertheless, don’t authors think that diagnosing Pf as Pv and vice versa has severe consequences?
19. Table 2A, from a total of 142 Pf infections (qPCR (>20 parasites/μL)), miLab correctly identified 33 of them, but misclassify 6 of them as negative and 6 as Pv. The issue of false negative and misdiagnosis looks significant. From a total of 64 Pv infections confirmed by qPCR, miLab correctly detects 50 of them as Pv, but misclassify 5 of them as negative and 9 of them as Pf infections. Of the 18 mixed infection by qPCR, miLab correctly identified 27.8% (5/18) of them while 5 of them as Pv only and 5 as Pf only.
