# Supplementary material for: A digital microscope for the diagnosis of Plasmodium falciparum and Plasmodium vivax, including P. falciparum with hrp2/hrp3 deletion
Source: PLOS Glob Public Health. 2024 May 20;4(5):e0003091. doi: 10.1371/journal.pgph.0003091 (PMC11104649; doi:10.1371/journal.pgph.0003091)
Supplement: Attachment — Submitted filename: Response_to_Reviewers.docx [file pgph.0003091.s006.docx]

We thank the reviewers for their detailed feedback. Please find our point-by-point responses below. Reviewer comments are in black, our answers are in blue.

Reviewer #1: Koepfli et al reports ‘A digital microscope for the diagnosis of Plasmodium falciparum and Plasmodium vivax, including P. falciparum with hrp2/hrp3 deletion’. Authors report malaria diagnostic challenges especially where multiple Plasmodium species are endemic. To address such challenges, the performances of a digital automated portable digital microscope from Noul MiLab were evaluated among 659 suspected malaria patients in Gondar, Ethiopia and 991 patients in Ghana. According to authors report miLab shows superior performance as compared to microscopy and comparable performance with RDTs. Authors also concluded that miLab was more sensitive than microscopy and thus is a valuable addition to the toolkit for malaria diagnosis, particularly for areas with high frequencies of hrp2/3 deletions.
Comments

1. The details of the performance of digital microscopy for the diagnosis of falciparum malaria in Ethiopia and Ghana were not well presented.

We feel results are clearly presented in the text, and in Table 1. Given this comment is very general, it is difficult to address it.

2. Authors didn’t indicate which specific regions of the hrp2/3 loci (exon 2, exon 1-2, upstream or downstream flanking regions) were genotyped to determine hrp2/3 gene deletion.

We have added the following information:

The *hrp2* assay amplifies a fragment in exon 2 immediately upstream of the histidine repeats that are detected by the RDT. This region of the gene is deleted among all samples were breakpoints were reported. The *hrp3* assay amplifies a fragment in the center of exon 2.

3. It’s unclear whether Noul MiLab, MiLab and digital microscope are interchangeably used as each appears at various places.

Noul is the manufacturer, and miLab is the name of the instrument. We have clarified this in line 80-81 as follows:
“The miLab, developed by Noul, is a fully integrated digital microscope for malaria diagnosis.”

We have only ever used the term ‘digital microscope’ to explain what the miLab is, such as in the abstract (“The Noul miLab is a fully integrated digital microscope for malaria diagnosis.”). At no point is the term ‘digital microscope’ used interchangeably with ‘miLab’.

4. The specificity of the miLab was 94.0%, which indicates the presence of ‘false positive’ results.

This is correct, false positive data result in lower specificity. It is unclear whether the reviewer recommends any change or whether this is a general observation.
5. The same RDTs were not used in Ethiopia and Ghana, hence, comparison of the RDT sensitivity seems challenging between the 2 countries.

This is correct. As explained in the methods section, the two studies were initially designed independently of each other. As a result, different RDTs were used. RDT data is provided separately for each country, at no point do we present RDT data that is combined for both countries.

We also clarified this in the methods section, please see answer to next comment.

6. Under MM section (line 116-117), it reads that ‘All samples were tested on site by miLab, and RDT’. I’m wondering why microscope wasn’t used onsite for malaria diagnosis given the study site is only 2,100meters away from Gondar town of Ethiopia?. Is this for Ghana or Ethiopia? Does the national policy of Ethiopia support this?

First, for clarification, the study site was at 2100 meters above sea level, not 2100 meters away from Gondar. To avoid any confusion, we have restructured the methods section and now first describe sampling in Ethiopia (including what RDT was used and how microscopy was conducted), followed by a description on sampling in Ghana. The text now reads (lines 111-146):

“In Ethiopia, the study was conducted in Maraki health center in the outskirts of Gondar. Gondar is a town of about 200,000 people located at approximately 2,100 meters above sea level. Malaria transmission is perennial, with a major peak from October to January, and a minor peak around May to July. Samples were collected during the main transmission season between November 1, 2021 and January 31, 2022. All individuals above one year of age and referred for testing for malaria were eligible to participate. In addition to blood collected for routine diagnosis by microscopy, approximately 150 µL blood was collected for diagnosis by miLab, RDT, and qPCR. All samples were tested on site by miLab, and RDT, and the result from the diagnosis conducted routinely by the local microscopist at the health center was recorded. In Ethiopia, samples were tested using the CareStart Malaria Pf/Pv(HRP2/pLDH) Ag combo RDT (lot MO19H71). At the time of the study, this test was used by the malaria control program. For qPCR, 50 µL blood was spotted on filter paper, air dried, packed into individual zip-lock bags, and stored at -20°C until extraction.

In Ghana, the study was conducted in Agona and Mankranso Government Hospitals near Kumasi where transmission is perennial. Samples were collected from January 26 to August 10, 2021. As in Ethiopia, all individuals above one year of age and referred for testing for malaria were eligible to participate, and an additional approximately 150 µL of blood was collected for this study. In Ghana, samples were screened with the Rapigen BIOCREDIT Malaria Ag Pf (pLDH/HRPII) RDT. This novel test has bands for HRP2 and LDH, and is more sensitive than RDTs that had been available previously (including the RDT used in Ethiopia) [5]. Expert microscopy has been considered the gold standard for parasite diagnosis and quantification for many decades [20]. As the protocol follows strict guidelines, expert microscopy data from different studies can be directly compared [21, 22]. For expert microscopy, thick and thin blood films were prepared and read by two expert microscopists. At least 100 high power fields were examined before adjudging slides as positive or negative. Parasite quantification was estimated based on the total number of malaria parasites counted per 200 or 500 white blood cells and then multiplied assuming 8000 white blood cells per µL blood [21]. For qPCR, whole blood was kept in 2 mL EDTA microtainers at -20°C until DNA extraction.

For miLab diagnosis in the health center in both countries, 200,000 RBCs were screened, corresponding to 0.04 µL blood. The miLab was operated on site, i.e. in the health center, either by local study personnel from University of Gondar or KNUST, or by health center personnel after a brief introduction. Operators of the miLab were not blinded, i.e., they were aware of the results of microscopy and RDT conducted at the health centers. A subset of slides from Ethiopia was further screened for 500,000 RBCs, corresponding to 0.1 µL blood. This screening was conducted in the Noul laboratory in South Korea.”

7. In line 180-181, it reads that ‘If a sample was positive by qPCR for P. falciparum but the miLab diagnosed possible P. vivax (or vice versa), it was counted as positive’. In country like Ethiopia where Pf and Pv are the two plasmodium species that co-exist, species identification is critical as the country has species-specific treatment policy. Does this give sense as far as miLab-based malaria diagnosis is concerned in Ethiopia?

We agree that species distinction of the miLab needs to be improved and added these data to the abstract:

“In Ethiopia, where *P. falciparum* and *P. vivax* were frequent, the miLab assigned the wrong species to 15/195 mono-infections at densities >20 parasites/µL by qPCR, and identified only 5/18 mixed-species infections correctly.”

To maintain consistency, we added the sensitivity >20 parasites/µL to the abstract.

In the discussion, we have clarified that misdiagnosis by microscopy is common in Ethiopia on lines 423-424:

“Similar levels of misdiagnosis by microscopy as in this study were reported from other sites in Ethiopia [27].”

Also in the discussion, we have put more emphasis on highlighting the implications of misdiagnosis (lines 426-432):

“In the case of misclassification of species, any positive patient will receive treatment, though guidelines differ, with artemether-lumefantrine recommended for *P. falciparum*, and chloroquine for *P. vivax*. Either treatment is expected to clear either species in many cases, and thus incorrect species diagnosis might be considered a lesser problem than false-negative results where patients remain untreated. Yet, misdiagnosis increases the risk of treatment failure due to drug resistance [28].”

8. Under data analysis (line 182-183), it reads that ‘the number of infections where the incorrect species was diagnosed, or mixed-species infections with only one species diagnosed, are presented separately’. It’s unclear why such data analysis is considered and authors shall provide details. The data analysis has to be made for all the infections regardless of whether they are correctly diagnosed or not.

Data for all infections is presented in Table 1 and the results section. The text above refers to an additional analysis focusing on samples where the wrong species was diagnosed by miLab. We have clarified this further as follows (line 285-286):

“In addition, the number of infections where the incorrect species was diagnosed, or mixed-species infections with only one species diagnosed, are presented separately.”

9. How authors did select the study sites in Ethiopia and Ghana?

The site in Ethiopia was selected because of the known prevalence of *P. falciparum* and *P. vivax*, which pose challenges for diagnosis. The site in Ghana was selected because of the known high level of *P. falciparum* transmission.

10. Did authors enroll every febrile patient who sought malaria diagnosis at both health centers of Ethiopia and Ghana? How authors account for the variation in sensitivity of DBS-based qPCR (in Ethiopia) vs whole-blood based PCR (Ghana) for malaria diagnosis?

As stated on lines 115 and 128, “all individuals above one year of age and referred for testing for malaria were eligible to participate.”

As we report sensitivity of the miLab vs. a threshold of 20 parasites/µL, the differences in overall LOD of DBS vs. whole blood-based qPCR are accounted for.

11. It is known that malaria is more endemic to Ghana than Ethiopia; however, it appears that the prevalence of malaria looks higher in Ethiopia (75.1% (495/659) individuals tested positive) than in Ghana (40.6% 402/991) tested positive) for P. falciparum. How authors justify this?

We can only report the data we collected, we cannot provide any justification and possible explanations remain speculative. It is possible that due to different levels of naturally acquired immunity, more infections in Ethiopia result in clinical symptoms, and thus a higher proportion of individuals test positive despite overall lower transmission rates. This is, however, pure speculation. It is not expected to affect results, thus we prefer not to discuss it in the manuscript.

12. Line 209-212, under ‘Sensitivity and specificity of P. falciparum and P. vivax diagnosis’. Is it possible for the authors to calculate the combined sensitivity of miLab (94.3% at densities >200 parasites/µL) for both Ethiopia and Ghana given Pv is uncommon in the latter? Or is the sensitivity of miLab for Pf only?

There is no P. vivax in our study site in Ghana. Table 1 includes sub-headers ‘P. falciparum’ and ‘P. vivax’. The combined data is presented under the sub-header ‘P. falciparum’

13. Although the sensitivity and specificity of miLab were calculated as shown in table 1; it’s unclear which tool(s) (qPCR or microscopy) was taken as a gold standard. Or do authors think/believe that miLab is superior to qPCR?

We have updated the legend of Table 1 as follows to clarify that we compared diagnostic tools to qPCR:

“Sensitivity and specificity for the diagnosis of *P. falciparum* and *P. vivax* compared to qPCR as gold standard. All density thresholds are based on qPCR data.”

14. It would be important to describe the total number of Pf and Pv samples subjected to calculate miLab sensitivity/specificity in table 1 so that readers can easily appreciate its diagnostic performances.

These numbers were already included in Table 1, but we realize it might not have been clear. We have updated the legend of Table 1 as follows:

“Numbers in brackets show number of positive or negative samples over total number of samples.”

15. The specificity of microscope and RDT is superior to that of miLab (table 1) in Ethiopia, how authors justify that miLab could lead to the treatment of uninfected individuals with antimalarial drugs? i.e from drug pressure, or drug resistance emergence/development perspectives?

We cannot comment/speculate on these issues, as they were not a subject of the current study.

16. Line 241-245, it’s indicated that ‘the miLab successfully diagnosed 51/52 (98.1%) infections with hrp2 deletion while field microscopy diagnosed 37/52 (71.2%) of hrp2-negative infections; what justification could authors provide for the inability of the microscope to detect of hrp2-deleted parasites while miLab did? Authors shouldn’t underestimate the issue of Pv misdiagnosis (5 infections) in Ethiopia.

Based on our data, sensitivity of field microscopy in Ethiopia was lower than miLab. We can only present the data we obtained in our study, we cannot speculate on why this is. It is unclear why the reviewer refers to Pv here, as this section deals with Pf only.

17. Line 238, when authors present ‘Diagnosis of hrp2/3 negative P. falciparum infections’, it would be imperative if they also provide ‘Diagnosis of hrp2/3 positive P. falciparum infections’ for readers to appreciate it.

Data for all samples is presented in Table 1, and in the main text.

18. Line 249-253, authors report that miLab has severe shortcomings in accurately diagnosing mixed infections (either the wrong species was assigned, or only one species was diagnosed in mixed species infections). Thus, in setting like Ethiopia where Pf and Pv are common and species-specific treatment policy exists, miLab diagnosis would lead to wrong drug regimens. Nevertheless, don’t authors think that diagnosing Pf as Pv and vice versa has severe consequences?

We have addressed this point in response to comment 7 above.

19. Table 2A, from a total of 142 Pf infections (qPCR (>20 parasites/μL)), miLab correctly identified 33 of them, but misclassify 6 of them as negative and 6 as Pv. The issue of false negative and misdiagnosis looks significant. From a total of 64 Pv infections confirmed by qPCR, miLab correctly detects 50 of them as Pv, but misclassify 5 of them as negative and 9 of them as Pf infections. Of the 18 mixed infection by qPCR, miLab correctly identified 27.8% (5/18) of them while 5 of them as Pv only and 5 as Pf only.

We have addressed this point in response to comment 7 above. For clarification, based on Table 2A, miLab correctly identified 130/142 Pf mono-infections (we are not sure where the 33 mentioned by the reviewer comes from).

Reviewer #2: Ewnetu et al. report results from a study to assess the diagnostic performance of the Noul miLab, a digital microscope for the diagnosis of malaria from whole blood. The manuscript is clearly written and should be accepted for publication once the minor issues detailed below are resolved.

1. Standardisation in reporting: Given that this is a study to assess diagnostic performance, the STARD checklist & flow-chart (<https://www.equator-network.org/reporting-guidelines/stard/>; BMJ 2015;351:h5527) need to be completed and appended as a supplementary item. In case it is found that the study does not conform to any of the items in the checklist (which does not, for the most part, appear to be the case for this manuscript), these should be addressed if appropriate as a limitation of the study in the discussion section. Further, it is important to note that the current recommendations for microscopy in research settings (see Malar J 2020;19:324) were not followed - it is acceptable to compare the performance against field microscopy but since 'expert' microscopy (more on that below) was performed in Ghana, it should have been possible to adhere to these recommended standards. The required information per the minimum reporting standards for malaria microscopy (including the criteria used to define 'expert' microscopy) should however be added to the relevant sections (see Am J Trop Med Hyg. 2021;104(3): 836–841).

We completed the STARD checklist. Given that all patients enrolled underwent all diagnostic tests (with the differences between Ethiopia and Ghana clearly explained, see response to Reviewer 1, point 6 above), we did not include a flow chart.

We added a few details that we had omitted to comply with the STARD guidelines, e.g.:

On blinding:
Lines 143-145: “Operators of the miLab were not blinded, i.e., they were aware of the results of microscopy and RDT conducted at the health centers.”

Lines 247-248: “Researchers conducting the qPCR were not blinded, i.e., they were aware of the results by local microscopy, expert microscopy, RDT, and miLab.”

On the reason for the refences tests:

Lines 132-134: “Expert microscopy has been considered the gold standard for parasite diagnosis and quantification for many decades [20]. As the protocol follows strict guidelines, expert microscopy data from different studies can be directly compared [21, 22].”

Lines 229-230: “qPCR can reach a limit of detection of <1 parasite per µL blood and was conducted as most sensitive assay to detect and quantify parasites [23].”

We agree about the comment on expert microscopy in Ethiopia. At the time of the study, no certified microscopist was available. This is made clear in the manuscript, and at no time do we combine field microscopy data from Ethiopia with expert microscopy data from Ghana.

2. Difference in specificity: There appears to be a significant difference in the specificity measured in Ethiopia vs Ghana. This would not be surprising if the device used manually stained slides given the notorious variability in slide quality. It is somewhat unexpected however to see this even with miLab cartridges which should protect the slides/captured images from any artefacts, often the cause of reduced specificity in image analysis-based diagnosis. The authors should clearly state whether or not there was any difference in or possibility of deviation from the recommended procedures followed at each of the sites and/or in the batches of cartridges used at the sites. At the very least this point needs to be added to the discussion section.

We agree that this difference is puzzling, and have added the following paragraph to the discussion (lines 434-438):

“A striking difference in specificity was observed between Ethiopia (specificity=86%) and Ghana (specificity=96%). This was surprising, as the use of the miLab prevents variation in slide quality, and the same algorithm was used for parasite detection. The relatively low number of only 164 true negative samples in Ethiopia might have reduced accuracy of specificity estimates in this site. Further, despite controls on data collection, eventual sample mix up at the health center cannot be fully ruled out.”

3. Statistics: There is no mention of sample size calculations - while it is not a major issue if none were performed, this should be clearly stated. The authors may also wish to include the kappa statistic (e.g., microscopy vs PCR and miLab vs PCR) for species detection in the results.

We added the following detail (lines 266-269):

“Limited data on the number of positive samples, and on the number of infections with densities >200 and >20 parasites/µL was available before the study. Thus, not formal sample size calculation could be conducted. The number of samples exceeds the number required to be able to detect a 5% difference in sensitivity with 95% confidence, which is 377 samples.”

While often used in similar studies, Kappa statistics is in fact the wrong tool to analyze our data. Kappa measures interrater reliability, i.e. whether two methods that are expected to yield the same result agree (e.g., it can be used to test whether two microscopists reading the same slides obtain the same results). We did not expect that miLab, RDT, microscopy, and qPCR had the same sensitivity, thus Kappa statistics should not be used. Using Kappa for example to compare PCR to an RDT results in puzzling data, as a less sensitive PCR will result in better agreement and a more sensitive PCR will reduce agreement (see here for a recent comment on differences in LOD of PCRs: https://malariajournal.biomedcentral.com/articles/10.1186/s12936-023-04822-w).

Other minor/typographical corrections:
Line 61: These RDTs also detect HRP3 which shares a highly similar sequence with HRP3 - correct this to HRP2.

Thank you for spotting this typo. We have corrected it.

Line 191: '...>20 parasites per uL..' should be >=20 parasites/uL?

We have clarified that “Samples with densities >0 to <=20 parasites/µL were excluded from AUC calculations”. There were no practical implications, as no sample had a density of exactly 20 parasites/uL.

Lines 242-3: '45/140 (32.1%) carried double deletions. The miLab successfully diagnosed 51/52 243 (98.1%) infections with hrp2 deletion (all except 3 also carried hrp3 deletions).' - please clarify/correct how many cases with both hrp2 & hrp3 deletions - 45 or 49?

Thank you for spotting this error. Samples with missing *hrp3* data had by mistake been counted as carrying the deletion. We have corrected this sentence as follows (lines 347-349):

“The miLab successfully diagnosed 51/52 (98.1%) infections with hrp2 deletion (45 also carried hrp3 deletions, 3 were wild type, and for 4 samples no hrp3 deletion typing data was available).”
